# Pertussis and influenza immunization: perceived attitude and decision of postpartum patients

**DOI:** 10.1186/s12884-022-05296-5

**Published:** 2022-12-28

**Authors:** Nutan B. Hebballi, Tayler Parker, Elisa I. Garcia, Dalya M. Ferguson, Susan Lesser, KuoJen Tsao, Maryam Broussard, Susan H. Wootton

**Affiliations:** 1 Department of Pediatric Surgery, McGovern Medical School at The University of Texas Health Science Center at Houston, 6410 Fannin St., Suite 471, Houston, TX 77030 USA; 2 Department of Internal Medicine, McGovern Medical School at The University of Texas Health Science Center at Houston, Houston, TX USA; 3 Children’s Memorial Hermann Hospital, Houston, TX USA; 4 Department of Pediatrics, McGovern Medical School at The University of Texas Health Science Center at Houston, Houston, TX USA

**Keywords:** Influenza, Maternal vaccination, Pertussis, Pregnancy, Prenatal vaccination, Postpartum, Whooping cough

## Abstract

**Background:**

Vaccination of pregnant patients with tetanus toxoid, reduced diphtheria toxoid, and acellular pertussis (Tdap) and influenza vaccine during influenza season can reduce maternal and fetal morbidity and mortality; nevertheless, vaccination rates remain suboptimal in this patient population. To investigate the effect of a brief educational counseling session on maternal Tdap and influenza vaccination and determine factors influencing women’s decision in regards to receiving Tdap and or influenza vaccine during their pregnancy.

**Methods:**

A face-to-face semi-structured cross-sectional survey was administered to postpartum patients on their anticipated day of discharge (June 11-August 21, 2018). A brief educational counseling session about maternal pertussis and Tdap vaccine was provided to interested patients after which the Tdap vaccine was offered to eligible patients who did not receive it during their pregnancy or upon hospital admission. Medical records were reviewed to determine if surveyed patients were vaccinated prior to discharge.

**Results:**

Two hundred postpartum patients were surveyed on their day of anticipated discharge. Of those who were surveyed, 103 (51.5%) had received Tdap and 80 (40.0%) had received influenza vaccinations prior to hospitalization. Among immunized patients, the common facilitators were doctor’s recommendation (Tdap: 68, 54.4%; influenza: 3, 6.0%), to protect their baby (Tdap: 57, 45.6%; influenza: 17, 34.0%) and for self-protection (Tdap: 17, 13.6%; Influenza: 17, 34.0%). Of the 119 participants who had not received either Tdap or influenza vaccine prior to the survey, the barriers cited were that the vaccine was not offered by the provider (Tdap: 36, 52.2%; influenza: 29, 27.6%), belief that vaccination was unnecessary (Tdap: 5, 7.2%; influenza: 9, 8.5%), safety concerns for baby (Tdap: 4, 5.8%; influenza: 2, 1.9%). Of 97 patients who were not immunized with Tdap prior to admission but were eligible to receive vaccine, 24 (25%) were vaccinated prior to survey as part of routine hospital-based screening and vaccination program, 29 (38.2%) after our survey.

**Conclusion:**

Interventions to educate pregnant patients about the benefits of vaccination for their baby, addressing patient safety concerns, and vaccine administration in obstetricians’ offices may significantly improve maternal vaccination rates.

## Background

Pertussis or whooping cough is a bacterial infection caused by *Bordetella pertussis* and Influenza is a viral infection that attacks the respiratory system. Both are highly contagious and spread from person to person through respiratory droplets. The burden from these respiratory infections is unevenly distributed, with infants being the highest risk group to experience morbidity and mortality [1, 2]. In 2018, 2031 cases of pertussis and 3 deaths in children age < 1 year were reported in the United States (US) [3]. Infants with pertussis are often undiagnosed and under-reported as they may not present with classic symptoms such as apnea [4]. During the 2019–2020 influenza season, 187 pediatric deaths were reported with 11 being children age < 6 months [5, 6]. Additionally, pertussis takes a significant toll of healthcare system. For infants aged 1 month and 7–12 months, the average incremental cost of pertussis ranged from $18,781 to $3772 respectively [1].

Vaccination of pregnant women with Tdap and influenza helps protects infants and reduces the risk of hospitalization. Maternal Tdap vaccination has been shown to be 58% effective in preventing hospitalization of infants infected with pertussis than infants born to unvaccinated mothers [7]. Likewise, infants of vaccinated mothers had a 45–48% lower risk of being hospitalized for influenza than infants of unvaccinated mothers [8].

To prevent pertussis and influenza infection among infants, the Advisory Committee on Immunization Practices (ACIP) recommends maternal pertussis vaccination between 27 and 36 weeks gestation to maximize passive antibody transfer to infants [9] as well as influenza vaccination during influenza season [10]. Women who are not vaccinated during their pregnancy are encouraged to get vaccinated against pertussis and influenza postpartum. Despite these widespread recommendations, vaccination among pregnant women is suboptimal. Among a national, opt-in internet panel sample of women who were pregnant during the 2017–18 influenza season, only 49.1% received influenza vaccine before or during their pregnancy, 54.4% of women with a live birth received Tdap vaccine and 32.8% received both vaccines [11]. Another internet panel survey conducted between March 31–April 16, 2021 to assess the end-of-season flu and Tdap vaccination coverage among women who were pregnant during the 2020–21 flu season reported that only 54.5% received influenza vaccine, 53.5% received Tdap with a live birth and 30.7% received both vaccines [12].

In 2010, a local surveillance monitoring system was established at our institution to improve maternal vaccination rates in our patient population. Since its inception, a monthly dashboard and daily report containing patient level data are generated and are securely emailed to front-line nursing managers to advise them of the vaccination rates and help vaccinate the unvaccinated patients prior to discharge. However, the dashboard and the daily reports were underutilized by front line nursing managers and nursing staff. Additionally, systematic counseling for mothers refusing vaccination was not a component of the surveillance system. Given these limitations, we implemented a quality improvement project to demonstrate that educating patients through brief counseling can improve Tdap vaccination rates. We had two objectives for this study: 1) to investigate the effects of brief educational counseling on vaccination rate of Tdap vaccine in postpartum patients and 2) to determine the factors influencing women’s decision regarding receiving Tdap and or influenza vaccine during pregnancy.

## Methods

### Setting and participants

This study was conducted at Children’s Memorial Hermann Hospital (CMHH) located at the Texas Medical Center in Houston, Texas. It was approved by The University of Texas Health Science Center at Houston institutional review board (HSC-MS-18–0390). Memorial Hermann is a non-profit healthcare system comprising of 14 hospitals and with > 24,700 deliveries in 2017 [13]. CMHH provides Level IV neonatal intensive care unit, the highest level of care to the premature and critically-ill babies born as early as 23 weeks [14]. We conducted a cross-sectional study using semi-structured surveys of postpartum patients between June 11 and August 21, 2018. Patients who were age ≥ 18 years, delivered viable babies, and spoke English or Spanish were eligible.

### Survey instrument

An electronic semi-structured survey was developed by the core research team consisting of pediatric surgeon, pediatrician with expertise in infectious disease, quality improvement nurse, research nurse, and research associate. The survey included questions on demographics, prenatal care, knowledge about Tdap and influenza vaccine, information around Tdap and influenza during prenatal care, and reasons of vaccinations or refusal/decline of Tdap and influenza vaccine. A combination of closed and open-ended questions to elicit qualitative data were used, and pilot tested in English with a convenient sample of postpartum patients and further revised to improve understandability and content before translating it into Spanish by a native Spanish speaker, who is also fluent in English. Both English and Spanish surveys were hosted on institutions’ secured REDCap database and were administered using an iPad.

### Survey administration

A member of the survey administration team contacted the postpartum unit nurse manager on weekdays (Monday–Friday) to obtain the list of postpartum patients that were to be discharged that day, and then informed the postpartum patients about the study. A convenience sample of patients who were interested to participate in the survey were included. Interested participants signed the consent form to complete the electronic survey and granted permission to review their electronic medical records. The semi-structured survey was administered face-to-face by one of the survey administration team members whilst another member recorded the participants’ responses along with their field notes on an iPad.

### Brief educational counseling

The survey administration team provided a brief educational counseling session to patients who were interested to learn about pertussis (whopping cough). This included the importance of caregivers getting vaccinated to protect the baby, safety, timing of the Tdap vaccine using the Centers for Disease Control and Prevention (CDC) infographic material [15]. A copy of the infographic material was provided to the patients. Tdap vaccine was then offered to eligible patients who had not received it during their pregnancy or as part of the hospital surveillance program and a bedside nurse was notified if the patient was willing to get vaccinated prior to discharge. Given that this study was conducted during the non-influenza season, brief educational counsel regarding the influenza vaccine was not provided to the patients. The median survey administration and brief educational counseling duration was approximately 20 min (range: 15–30 min).

### Medical record review

We reviewed the electronic medical records of survey participants to determine if the bedside nurses had completed the electronic vaccination screening forms as part of their routine clinical practice and if eligible patients received the Tdap vaccination prior to their discharge from the hospital.

### Data analysis

Descriptive analysis was conducted for all survey responses. Categorical variables were summarized using frequency distribution and were reported as number and total percentage. Continuous variables were reported as a mean with standard deviation. For open-ended questions that described patient’s related factors that influenced their decision of receiving the Tdap and influenza vaccine were grouped into barriers and facilitators based on the emerging themes. Stata version 15 was used for all quantitative analyses (StataCorp, College Station, TX).

## Results

We surveyed 200 postpartum patients over 10 week’s period on their anticipated day of discharge. Most participants were English-speaking (185, 92.5%), African-American (87, 54.7%), Non-Hispanic (116, 63.7%), had Medicaid (117, 58.5%) and were married or lived with a partner (129, 64.5%) (Table 1). Most received 12 grade education or lower (72, 36.0%), had prenatal care (192, 96.0%) and were multiparous (111, 55.5%) (Table 1).

**Table 1:** Characteristics of patients enrolled in the study

Characteristics	Patients (*N* = 200) *
**Age in years**	28.2 ± 6.02
**Estimated gestational age (in weeks)**	37.1 ± 3.3
**Preferred language**	
English	185 (92.5)
Spanish	7 (3.5)
Other	8 (4)
**Race**	
Caucasian	52 (32.7)
African-American	87 (54.7)
Asian/Pacific Islander	15 (9.4)
Native American	2 (1.3)
Mixed Race	3 (1.9)
**Ethnicity**	
Hispanic	66 (36.6)
Non-Hispanic	116 (63.7)
**Education**	
12^th^ grade or lower	72 (36)
Some college or associate degree	64 (32)
Bachelor’s or postgraduate degree	52 (26)
No post graduate degree	2 (1)
**Marital Status**	
Married	95 (47.5)
Single	70 (35)
Single living with partner	34 (17)
Insurance**	
Private	85 (42.5)
Medicaid	117 (58.5)
Medicare	14 (7)
Other (Military or Veteran)	4 (2)
**Multiparous**	111 (55.5)
**Received prenatal Care**	192 (96)

*SD* Standard deviation

*n* = total number represents the total number of participants who provided a response

^*^ Characteristics summarized as n (%) or mean ± SD

^**^ Includes combinations of insurances

Most patients received Tdap (103, 51.5%) or influenza (80, 40.0%) vaccinations prior to delivery with most having been vaccinated at their obstetrics or physician’s office (Tdap: 88, 70.4%; influenza: 50, 62.5%). Additional sites for prenatal vaccination included pharmacies (Tdap: 12, 9.3%; influenza: 10, 12.5%), during prior hospitalizations (Tdap: 25, 19.4%; influenza: 8, 10%), and at other locations such as church or work site (influenza: 12, 15%).

The knowledge received by participants about Tdap and influenza vaccines at their obstetrics or physician’s office differed. A total of 103 (83.1%) participants reported receiving information about Tdap whereas 50 (25%) received information about influenza vaccination. Only 65 (63.1%) patients received handouts related to Tdap and 15 (30%) received handouts related to influenza vaccine. Only 74 (71.8%) patients discussed Tdap and 33 (66.0%) discussed influenza vaccine with their physicians or nurse practitioner. Lastly, 11(10.7%) participants discussed Tdap and 4 (8%) influenza vaccination with the provider’s office staff.

The emerging themes of the factors that influenced maternal vaccinations among participants were grouped into perceived facilitators and barriers (Table 2). For Tdap, the most influencing factor was a health care provider’s (HCP) recommendation (68, 54.4%), to protect their baby (57, 45.6%) and to protect themselves along with their baby (17, 13.6%). Participants who received an influenza vaccine during the influenza season cited protecting their baby (17, 34%) and self-protection (17, 34%) were the most influencing factors, followed by mandatory for work (5, 10%) and on HCP recommendation (3, 6%). Regarding barriers, the most common reason was not being offered by a HCP (Tdap (52.2%) and influenza vaccine (27.6%)). Other perceived barriers included the belief that vaccine was unnecessary (Tdap: 7.2%; influenza: 8.5%), safety concerns for their baby (Tdap: 5.8%; influenza: 1.9%).

**Table 2:** Themes of perceived facilitators and barriers to Tdap and influenza vaccines

**Tdap Vaccine**	
**Facilitators**	**Barriers**
**Theme 1: Recommendation by healthcare provider**	**Theme 1: Not offered by healthcare provider**
“Doctor recommended it to them, knew it was important”	“Not offered by her OB. Didn’t know anything about pertussis or what the shot was”
“She works in a daycare and her doctor recommended that she get it so she was following the recommendation”	“It wasn't offered to her. She had never heard of it before at all.”
**Theme 2: For infant’s protection**	**Theme 2: Vaccine unnecessary**
“I'm a nurse so they may not have given me information or explained to me why I needed it. I decided to get it because it's to protect the baby”	“Not offered to her. She said she was familiar with the vaccine and didn't think she needed it.”
“She got it for prevention for the baby. She had also seen some commercials on TV talking about pertussis and how it's important to protect the baby”	“At first she couldn't remember why she hadn't had it but then she said it's because she didn't think it was necessary for her to get.”
**Theme 3: For self-protection**	**Theme 3: Risk to the baby**
“Protect me and the baby”	“It was offered. She didn't get it because it was optional and she considered it to be high risk for the baby.”
	“She was scared of vaccines because she said she heard some knowledge and research that vaccinations were bad. She didn't want to hurt baby or herself.”
**Influenza Vaccine**	
**Facilitators**	**Barriers**
**Theme 1: For infant’s protection**	**Theme 1: Not offered by healthcare provider**
“They said that the flu could be especially dangerous for the baby because they are more vulnerable.”	“Not offered. Has gotten it in the past but didn't realize she needed to get it.”
**Theme 2: For self-protection**	**Theme 2: Become sick from influenza vaccine**
“The vaccine will help you not get sick, especially since it's the flu season”	“She had gotten it in the past and felt funny afterwards. She was scared that her or her kids would get sick.”
**Theme 3: Required for work**	**Theme 3: Risk to the baby**
“Got it before pregnancy. Required for work every year.”	“She was scared since it is a virus and she was worried it would risk hurting the baby”
“Had the shot at work because it was required”	“She was afraid it would hurt baby. She said she was trying to be a protective mom”

For 158 (79.0%) participants, the self-reported vaccination information for Tdap matched the medical record documentation and 16 (8.0%) received Tdap vaccine prenatally although there was no record of vaccination in the hospital vaccination screening form. Also, 97 (48.5%) postpartum patients did not receive their Tdap vaccine prior to the hospital admission but were eligible for vaccination. There were 24 (25%) participants vaccinated as part of our hospital surveillance monitoring program prior to completing the survey and receiving the Tdap educational counseling. Upon completion of the survey and receiving the brief educational counseling on Tdap vaccine, 29 (38.2%) postpartum patients received their Tdap vaccine prior to discharge, and 22 (22.0%) were never vaccinated.

## Discussion

Our survey of 200 postpartum participants who agreed to participate in an educational counseling session demonstrated that 38.2% who were previously unvaccinated during pregnancy, received Tdap vaccine prior to hospital discharge after receiving brief educational counseling, indicating that educational counseling can improve vaccination rates for Tdap in pregnancy beyond routine surveillance programs. Additionally, our study demonstrated that the most important factors influencing participants to receive Tdap and influenza vaccination was the desire to protect their baby, protect themselves and HCP recommendation, which are consistent with factors noted in other global studies [16–18].

Only half of our participants received Tdap vaccine and less than half received influenza vaccine prenatally, highlighting a gap in maternal vaccination coverage. These findings are consistent with national immunization rates for the same duration as our study. In April 2019, the maternal vaccination coverage for Tdap was 54.9% and 53.7% for influenza [19], and 53.5% for Tdap and 54.5% for influenza in April 2021 [20]. This is despite recommendations from ACIP and CDC to vaccinate pregnant patients. Successful strategies to improve prenatal vaccination rates have included policy recommendations, community-based interventions such as implementation of a community preventive task force, and family incentives for vaccinations [21]. Additional strategies include cocooning that has helped achieve over 90% vaccination rates for Tdap and over 90% rates for influenza vaccines in postpartum patients [22, 23]. However, these programs did not include one-on-one counseling of patients as we implemented in our study. Given Tdap vaccination of pregnant patients reduces infant hospitalizations (38% vs 32%), infant death (49% vs 29%) and saves cost per adjusted life-year compared to cocooning ($414,523 vs $2,005,940) [24], educational counseling is a worthwhile strategy to increase vaccination uptake.

Although national surveillance of Tdap and influenza vaccines are carried out by CDC annually, very little monitoring occurs at the hospital/organization level. This can be attributed to barriers such as staff shortages, ambiguous guidelines, vaccine purchase and storage, and reimbursements for vacciantions [25]. In our institution, a paper-based Tdap screening form was replaced by an electronic screening form in 2010 which is now routinely used for all adult and pregnant patients [22]. Despite the use of an electronic Tdap screening form, an automated vaccine tracking system and dashboard, vaccination rates of Tdap and influenza remained suboptimal in pregnancy. Although suboptimal, our study had a major impact in increasing Tdap vaccination for postpartum patients from essentially zero to 29 (38.2%) patients demonstrating that brief patient counseling using semi-structured surveys and education as a cocooning strategy at the hospital can open up discussions with patients regarding the effects of Tdap vaccine, their benefits during and after pregnancy, and address any safety concerns postpartum patients may have regarding Tdap vaccine prior to discharge. Additionally, brief educational counseling is a low-cost intervention and does not take up much of the nursing staff time, who are already busy with clinical duties and patient care. Moreover, using an educational counseling approach, nurses can be easily trained regarding how to respond to patients’ refusals and reinforce the safety and effectiveness of maternal vaccination. In fact studies have shown that having a nurse-driven vaccination program and having a designated vaccination nurse serving as clinical champion at the hospital, who can regularly brief unit nurses about vaccinations during their shift changes, encourage staff to educate patients about vaccination, answer questions regarding vaccinations throughout patient’s hospitalization, and monitor weekly monitor vaccination rates among pregnant patients can increase Tdap and influenza vaccine vaccination rates [26, 27].

Our participants who chose to receive Tdap and influenza vaccines appeared to have had adequate knowledge about the vaccine, based on recommendations from their providers, to protect their baby and for self-protection. Likewise, participants who did not receive these vaccines prenatally cited the lack of recommendations by providers, safety concerns for their baby and for themselves. These findings are consistent with other studies that demonstrated healthcare providers are more likely to recommend Tdap vaccination to their pregnant or postpartum patients’ if there are strong national recommendations by ACIP and American College of Obstetricians and Gynecologists (ACOG) [28]. Studies have also shown that pregnant patients who have accurate information towards the safety and effectiveness of vaccine are more likely to be vaccinated indicating the importance of prenatal vaccine related education [29–31]. Further research is needed to determine if providing vaccine related education early on during the pregnancy and offering vaccinations at the HCP office during patient’s routine third trimester visit for Tdap will alter their perceptions and improve patient acceptance rates of the vaccine.

There are several limitations of this study. First, our study participants consisted of a convenience sample of postpartum patients, which may have introduced a selection bias in our results. Second, we included women who spoke English and Spanish in our study and thus we cannot conclude that the facilitators and barriers mentioned in this study will be similar for women who spoke other languages. Third, there is a possibility of recall and reporting bias that might have influenced some of our results. Finally, our participants came from a specific geographic location from a single hospital setting, we had higher proportion of African-American participants and only 36% of our participants received high school or lower education potentially limiting the generalizability of our results to other geographic regions, other races and educational level. The biggest strength of this study is we validated the Tdap vaccination received by postpartum patients’ who were willing to receive vaccination after our survey administration.

## Conclusion

To improve maternal vaccination rates, it is imperative to implement educational and administration interventions for HCPs and pregnant women, specifically addressing the benefits and safety of maternal vaccination. Given that Tdap and influenza vaccinations in pregnancy reduce infant hospitalizations, infant deaths and saves cost per adjusted life-year compared to other strategies, educational strategies are worthwhile in increasing uptake in pregnancy.

## Data Availability

The datasets generated during and analyzed during the current study are not publicly available due to restrictions but are available from the corresponding author on reasonable request.

## Abbreviations

ACIP: Advisory Committee on Immunization Practices
ACOG: American College of Obstetricians and Gynecologists
CDC: Centers for Disease Control and Prevention
CMHH: Children’s Memorial Hermann Hospital
Tdap: Tetanus toxoid, reduced diphtheria toxoid, and acellular pertussis
